# Association of metabolic syndrome and frailty with postoperative complications in older gastric cancer patients: A body composition perspective

**DOI:** 10.1002/cam4.70194

**Published:** 2024-09-24

**Authors:** Xiaoman Jiang, Lingyu Ding, Yinning Guo, Xueyi Miao, Kang Zhao, Li Chen, Shuqin Zhu, Xinyi Xu, Qin Xu

**Affiliations:** ^1^ School of Nursing Nanjing Medical University Nanjing China; ^2^ Department of Gastrointestinal Surgery the First Affiliated Hospital of Zhejiang University, School of Medicine Hangzhou China; ^3^ Department of General Surgery the First Affiliated Hospital of Nanjing Medical University Nanjing China; ^4^ Faculty of Health Queensland University of Technology Brisbane Queensland Australia

**Keywords:** frailty, gastrointestinal cancer, metabolic syndrome, postoperative complications, surgery

## Abstract

**Objectives:**

To compare the characteristics of body compositions between metabolic syndrome (MetS) and frailty, and determine the independent and overlapping of MetS and frailty with postoperative complications among older patients with gastric cancer.

**Design:**

A prospectively observational study.

**Setting and Participants:**

Two hundred and eighty six older patients from 60 to 80 years undergoing radical gastrectomy for the first time.

**Measurements:**

MetS was diagnosed by the criteria from the 2020 edition of Chinese guideline for the prevention and treatment of type 2 diabetes mellitus, and frailty was defined by frailty phenotype. An InBody770 impedance analyzer was used to measure body compositions and with 10 fat‐ and muscle‐related indicators being included in this study. Based on the presence of frailty and MetS, patients were divided into the frailty group, MetS group, frailty+MetS group, and normal group, and the body compositions indicators of these groups were compared. Clavien–Dindo classification was used to grade the severity of postoperative complications. Univariate and multivariate regression models were performed to explore the independent and joint association of MetS and frailty with postoperative complications.

**Results:**

The incidence rate of MetS, frailty, and frailty+MetS being 20.3%, 15.7%, and 4.2% respectively. Compared with the normal group, both fat and muscle compositions were decreased significantly in the frailty group (*p <* 0.05), while the statistically significant difference of fat‐to‐muscle mass ratio (FMR) and skeletal muscle mass to visceral fat area ratio (SVR) were not observed (*p* > 0.05). In contrast, except SVR, the other indicators of the MetS group were higher than the normal group (*p <* 0.05). As to the frailty+MetS group, there was a significant increase in fat compositions and FMR, as well as a significant decline in SVR (*p <* 0.05), while the difference of muscle compositions was not statistically significant (*p* > 0.05). There was an association of frailty with postoperative total (OR = 3.068, 95% CI: 1.402–6.713) and severe (OR = 9.423, 95% CI: 2.725–32.589) complications, but no association was found of MetS alone. MetS coexisting with frailty was associated with the highest risk of both total (OR = 3.852, 95% CI: 1.020–14.539) and severe (OR = 12.096, 95% CI: 2.183–67.024) complications.

**Conclusions:**

Both frailty and MetS coexisting with frailty had adverse effects on postoperative complications, which appeared greatly different characteristics in body compositions and therefore reinforced the importance of targeted nutritional or metabolic intervention. Although MetS alone were not significantly associated with postoperative complications, it is essential to focus on the causal relationship and development trend between MetS and frailty to prevent MetS from shifting into frailty, considering the highest risk in their coexistence state.

## 1 INTRODUCTION

Gastric cancer, one of the most prevalent malignancies globally, ranks fifth in incidence and fourth in mortality rates. In China, it accounts for 43.9% of new cases and 48.6% of deaths.^1^ Notably, the older experience a higher incidence, with over 70.8% of gastric cancer patients in China being over 60 years old.^2^ Due to the accumulation of physiological damage with aging, as well as the abnormal nutritional metabolism caused by tumor‐related digestion and absorption disorders, the postoperative complication has been a serious problem among these population, which could go as high as 41.4%.^3^ As postoperative complication is a much important evaluation factor for perioperative rehabilitation and is closely related to long‐term prognosis,^4^ it is essential to early screen out high‐risk population and implement effectively targeted intervention among older patients with gastric cancer.

Both frailty and metabolic syndrome (MetS) are crucial aging‐related concerns, and the former represents a state of dysfunction of multiple systems and decrease of physiological reserves,^5^, ^6^ while the latter is a clinical cluster of abdominal obesity, hyperglycemia, hypertension, and dyslipidemia.^7^ Our previous study has demonstrated that preoperative frailty was an important risk factor for adverse postoperative outcomes among digestive cancer patients,^8^ and MetS was significantly associated with the presence of frailly.^9^ However, although MetS has been proven to be closely related to the genesis and development of cancer, its effect on postoperative outcomes among cancer population is still controversial, with some studies suggesting a protective effect on overall survival in older patients with gastric cancer.^10^, ^11^ Other studies also suggested that frailty, not MetS, was associated with mortality in older people.^12^, ^13^ Given the similarity between MetS and frailty in pathophysiology and high prevalence among the older,^14^ MetS and frailty may have additive or synergistic effects on postoperative outcomes, while very few studies have concerned this point.

Moreover, considering body composition features, decrease in muscle mass and function could be the core of frailty,^15^ while the accumulation and abnormal distribution of adipose could be the core of MetS.^16^ However, recent studies showed that obesity‐related indicators, such as body mass index (BMI) and waist circumference were closely related to frailty, and there might exist “obesity paradox” among frail people.^17^ For example, Watanabe et al. found that frailty could modify the association of BMI with mortality among older adults, and frail people may benefit from a higher BMI compared to those non‐frail people.^18^ Also, studies found people with MetS may experience reduction in muscle mass and be characterized by sarcopenic obesity.^19^, ^20^ In addition to single muscle‐ or fat‐related indicators, the ratio of both have been recently found to be of great value for predicting long‐term survival and mortality among cancer patients.^21^, ^22^ However, to the best of our knowledge, no previous studies have explored body compositions characteristics specific to frailty and MetS among older patients with gastric cancer. It is well known that body composition could reflect nutritional status and greatly impact peri‐operative outcomes among gastric cancer patients^23^; therefore, we hypothesized that understanding these characteristics could help explain effects of frailty and MetS on postoperative complications.

Therefore, the aim of this study was to contribute to the literature by investigating body composition characteristics specific to frailty and MetS among older gastric cancer patients, and determine whether the presence of MetS and frailty, independently or in combination, affect postoperative complications among these population.

## 2 METHODS

### 2.1 Study design and participants

This prospectively observational study was carried out at the first affiliated hospital of Nanjing Medical University, and the convenience sampling method was used to continuously include older patients admitted to general surgery department for gastric cancer from August 2021 to August 2022. The included participants were 60 to 80 years,^9^ as well as being first diagnosed with gastric cancer confirmed by endoscopy and biopsy and underwent radical gastrectomy. The excluded participants were as follows: (1) unable to complete all the measurement due to serious physical, cognitive or mental impairment, such as limp due to diabetic foot, “Alzheimer's Disease”, or depression; (2) suffered serious infectious diseases or other severe conditions, such as heart failure and maintenance hemodialysis; (3) combined with other site of malignant tumors; (4) implanted with metal medical devices such as cardiac pacemaker, or took medications that affect body composition measurements; and (5) received neoadjuvant therapy before surgery.^24^ During the study period, researchers assessed whether each newly admitted patient met the above criteria, and then explained the purpose, benefits, and risks of the study through face‐to‐face communication to increase patients' trust and knowledge of this study. Patients who met the criteria and were willing to participate were recruited. Patient information were input and updated through establishing an Excel spreadsheet, and researchers timely followed up to ensure the successful progress of the research project.

Our pre‐survey result of the first 3 months showed that the incidence of postoperative total complications among these patients was about 20%, and with the confidence interval and tolerance error being 0.95 and 0.10 respectively, the sample size calculated by PASS 11.0 software was 264 cases. Considering the loss rate of 5%–10%, at least 277 cases should be included in this study. This study was approved by the Ethics Committee of Nanjing Medical University and was conducted in accordance with the Declaration of Helsinki. All procedures were carried out with the adequate understanding and written consent of the participants. This study was registered with the Chinese Clinical Trial Registry on June 5, 2022, with the clinical trial number being ChiCTR2200060615.

### 2.2 Baseline variable

The baseline variables were designed as a standardized questionnaire and were collected through face‐to‐face interview with participants or electronic medical records. After participants' admission, age, sex, BMI, comorbidity (comorbidity was defined as the concurrent presence of two or more medically diagnosed diseases in the same individual, such as hypertension, diabetes, hyperlipidemia and stable coronary artery disease), drug intake (polypharmacy was defined as >4 chronic drug use), history of smoking, history of drinking, history of abdominal surgeries, nutritional risk screening 2002 (NRS2002 score), serum albumin concentration (hypoalbuminemia was defined as <35 g/L), and hemoglobin concentration (anemia was defined as <130 g/L in male or <120 g/L in female) were collected.^25^ After the participants had undergone surgery, pathologic tumor‐node‐metastasis (pTNM) stage according to the TNM staging of the 8th edition of American Joint Committee on Cancer (AJCC), operation method (open or laparoscopic surgery), operation type (total, distal subtotal, or proximal subtotal gastrectomy), tumor size (<5 cm or ≥5 cm), tumor differentiation (low, moderate or high) was collected via electronic medical records.

### 2.3 MetS criteria

The diagnostic criteria for MetS used in this study was from the 2020 edition of Chinese guideline for the prevention and treatment of type 2 diabetes mellitus,^26^ which requires any three or more of the following five criteria: (1) central obesity (waist circumference ≥90 cm for male and ≥85 cm for female); (2) elevated blood glucose (fasting blood glucose ≥6.1 mmol/L and (or) blood glucose of 2 h after glucose loading ≥7.8 mmol/L and (or) diagnosed diabetes with treatment); (3) elevated blood pressure (systolic blood pressure ≥130 mm Hg and (or) diastolic blood pressure ≥85 mm Hg or diagnosed hypertension with treatment); (4) elevated triglycerides (TG) ≥1.7 mmol/L; (5) reduced high‐density lipoprotein cholesterol (HDL‐C)<1.04 mmol/L.

### 2.4 Frailty criteria

Frailty phenotype proposed by Fried et al. in 2001^5^ was used in this study, and participants who met any three or more of the five criteria were defined as frailty. The specific five criteria were as follows: (1) weight loss (unexpected weight loss >4.5 kg or >5% of original body weight during the last year); (2) fatigue (for 3 days or more during the last week the participant reported that “I felt that everything I did was an effort” and/or “I could not get going” according to the Chinese version of Center for Epidemiological Studies‐Depression 10 (CES‐D10) scale)^27^; (3) decreased physical activity (assessed by the Short version of the International Physical Activity Questionnaire‐Chinese version (IPAQ‐S‐C))^28^; (4) slowness (determined by walking speed of 4.57 m classified by different gender and height); (5) low handgrip strength (determined by dominant handgrip strength measured by an electronic hand dynamometer (EH101, Guangdong Province, China) classified by different gender and BMI). The detailed evaluation process and criteria were from Chinese Expert Consensus on Frailty Management.^29^

### 2.5 Body compositions

Body compositions were measured by two trained researchers in strict accordance with the instructions of a direct, segmental, multi‐frequency bioelectrical impedance analyzer (InBody770；Biospace, Seoul, Korea). Participants were required to fast for solids and liquids for at least 2 h, and to empty urine and feces before measurement. Moreover, they should wear light clothes and remove metal accessories before measuring. Their arms should be straight and open, and the torso should be at an angle of about 15° as well as keeping a standing position.^30^ The specific indictors included body fat mass (BFM), fat mass index (FMI), percent body fat (PBF), visceral fat area (VFA), skeletal muscle mass (SMM), skeletal muscle mass index (SMI), appendicular skeletal muscle mass index (ASMI), fat‐to‐muscle mass ratio (FMR), and skeletal muscle mass to visceral fat area ratio (SVR). Among these, FMR was the ratio of BFM to SMM^31^ and SVR was the ratio of ASMI to VFA,^32^ which were calculated by researchers, and the values of other indicators were automatically derived from the electronic system.

### 2.6 Postoperative complications

According to Clavien–Dindo classification (CDC),^33^ the severity of postoperative complications was graded from I to V, with total complications graded as CDC ≥ II and severe complications graded as CDC ≥ III. The screened domains of postoperative complications contained pulmonary, infectious, cardiovascular, neurological, renal, gastrointestinal, wound, pain, and hematological complications.^34^

### 2.7 Statistical analysis

SPSS26.0 software was used to conduct statistical analysis. The Kolmogorove–Smirnov test was used to verify the normality of the continuous data distribution. Mean and standard deviation (SD) were used to describe measurement data that were normally distributed, and the median and interquartile range (IQR) were used to describe measurement data that were not normally distributed. Counting data were described by frequency and percentage. The measurement data conforming to normal distribution and homogeneity of variance were compared among multiple groups by one‐way ANOVA, and pair comparison was performed by LSD method; otherwise, Kruskal–Wallis rank sum test and Mann–Whitney *U*‐test were applied. Chi‐square test was used for comparing counting data among multiple groups. Taking postoperative complications as dependent variables, univariate Logistic regression analysis was performed to screen the potential risk factors, and factors with *p* < 0.10 were further included in the multivariate logistic regression analysis by a forward stepwise selection methodology. Variance inflation factor (VIF) was used to test multicollinearity in each multivariate logistic regression model. Statistical significance was set at *p* < 0.05 in this study, and VIF >5 was supposed to be with multicollinearity.

## 3 RESULTS

### 3.1 Baseline characteristics

A total of 412 hospitalized patients were recruited, of which 24 cases were excluded due to transfer to other departments before surgery, 52 cases were excluded for discharge without surgery, 9 cases were excluded for serious infectious or severe conditions, and 41 cases were excluded due to non‐radical gastrectomy. Finally, 286 participants were selected into this study. Based on the presence of frailty and MetS, patients were divided into four groups: the frailty group (*n* = 45), the MetS group (*n* = 58), the frailty + MetS group (*n* = 12), and the normal group (*n* = 171). Statistically significant differences were observed in terms of age, BMI, comorbidities, NRS2002, hemoglobin, albumin, and postoperative complications among the four groups (*p* < 0.05), while there were no statistically significant differences in other indexes (*p* > 0.05). Detailed information is summarized in Table 1.

**TABLE 1:** Characteristics of general information among four groups (mean ± SD/*n* (%)).

Variables	Frailty *n* = 45, 15.7%	MetS *n* = 58, 20.3%	Frailty+MetS *n* = 12, 4.2%	Normal *n* = 171, 59.8%	*X* ^2^ (*F*) value	*p* value
Age years mean ± SD	71.56 **±** 5.6	69.03 **±** 5.2	69.25 **±** 5.6	68.61 **±** 5.1	7.751	<0.001
Gender						
Male	36 (80%)	50 (86.2%)	7 (58.3%)	131 (76.6%)	5.133	0.162
Female	9 (20%)	8 (13.8%)	5 (41.7%)	40 (23.4%)		
BMI kg/m^2^						
Normal 18.5 ~ <24.0	29 (64.4%)	14 (24.1%)	3 (35%)	95 (55.6%)	55.802	<0.001
Low <18.5	8 (17.8%)	0 (0%)	1 (8.3%)	12 (7%)		
Overweight 24 ~ <28	6 (13.3%)	26 (44.8%)	3 (25%)	48 (28.1%)		
Obese ≥28	2 (4.4%)	18 (31%)	5 (41.7%)	16 (9.4%)		
Comorbidity						
No	25 (55.6%)	5 (8.6%)	1 (8.3%)	108 (63.2%)	60.279	<0.001
Yes	20 (44.4%)	53 (91.4%)	11 (91.7%)	63 (36.8%)		
Polypharmacy						
No	44 (97.8%)	52 (89.7%)	11 (91.7%)	165 (96.5%)	5.209	0.121
Yes	1 (2.2%)	6 (10.3%)	1 (8.3)%	6 (13.5)%		
NRS2002 score ≥3						
No	28 (62.2%)	49 (84.5%)	9 (75%)	146 (85.4%)	11.61	0.009
Yes	17 (37.8%)	9 (15.5%)	3 (25%)	25 (14.6%)		
History of smoking						
No	20 (44.4%)	30 (51.7%)	6 (50%)	107 (62.6%)	5.96	0.114
Yes	25 (55.6%)	28 (48.3%)	6 (50%)	64 (37.4%)		
History of drinking						
No	26 (57.8)%	27 (46.6%)	6 (50%)	105 (61.4%)	4.18	0.239
Yes	19 (42.2%)	31 (53.4%)	6 (50%)	66 (38.6%)		
History of abdominal surgeries						
No	42 (93.3%)	45 (77.6%)	12 (100%)	138 (80.7%)	7.657	0.054
Yes	3 (6.7%)	13 (22.4%)	0 (0%)	33 (19.3%)		
Serum albumin g/L						
Normal	26 (57.8%)	54 (93.1%)	9 (75%)	124 (72.5%)	17.535	0.001
Low	19 (42.2%)	4 (6.9%)	3 (25%)	47 (27.5%)		
Hemoglobin g/L						
Normal	12 (26.7%)	33 (56.9%)	5 (41.7%)	85 (49.7%)	10.380	0.016
Low	33 (73.3%)	25 (43.1%)	7 (58.3%)	86 (50.3%)		
pTNM						
I	12 (26.7%)	20 (34.5%)	4 (33.3%)	61 (35.7%)	8.73	0.183
II	15 (33.3%)	14 (24.1%)	0 (0%)	50 (29.2%)		
III	18 (40%)	24 (41.4%)	8 (66.7%)	60 (35.1%)		
Tumor size						
<5 cm	26 (57.8%)	44 (75.9%)	5 (41.7%)	118 (69%)	7.35	0.062
≥5 cm	19 (42.2%)	14 (24.1%)	7 (58.3%)	53 (31%)		
Tumor differentiation						
Low	20 (44.4%)	19 (32.8%)	5 (41.7%)	61 (35.7%)	6.54	0.339
Moderate	21 (46.7%)	35 (60.3%)	4 (33.3%)	96 (56.1%)		
High	4 (8.9%)	4 (6.9%)	3 (25%)	14 (8.2%)		
Operation type						
Total gastrectomy	27 (60%)	35 (60.3%)	8 (66.7%)	97 (56.7%)	3.577	0.719
Distal subtotal	17 (37.8%)	17 (29.3%)	4 (33.3%)	62 (36.3%)		
Proximal subtotal	1 (2.2%)	6 (10.3%)	0 (0%)	12 (7%)		
Operation method						
Open surgery	8 (17.8%)	9 (15.5%)	2 (16.7%)	20 (11.7%)	1.43	0.698
Laparoscopic surgery	37 (82.2%)	49 (84.5%)	10 (83.3%)	151 (88.3%)		
Postoperative total complications (CDC ≥ II)						
No	28 (62.2%)	49 (84.5%)	6 (50%)	146 (85.4%)	17.017	0.001
Yes	17 (37.8%)	9 (15.5%)	6 (50%)	25 (14.6%)		
Postoperative severe complications (CDC ≥ III)						
No	36 (80%)	54 (93.1%)	9 (75%)	166 (97.1%)	18.366	<0.001
Yes	9 (20%)	4 (6.9%)	3 (25%)	5 (2.9%)		

Abbreviations: BMI, body mass index; NRS2002, nutritional risk screening 2002; pTNM, pathologic tumor‐node‐metastasis; SD, standard deviation.

### 3.2 Comparison of body compositions

Compared with the normal group, weight, BMI, BFM, FMI, PBF, VFA, SMM, SMI, and ASMI decreased significantly in the frailty group (*p <* 0.05), while the statistically significant difference of FMR and SVR were not observed (*p* > 0.05). In contrast, except SVR, the other indicators of the MetS group were higher than the normal group, with the difference being statistically significant (*p <* 0.05). As to the frailty+MetS group, there was a significant increase in weight, BMI, BFM, FMI, PBF VFA, and FMR, as well as a significant decline in SVR (*p <* 0.05), while the difference of height, SMM, SMI, and ASMI were not statistically significant compared with the normal group (*p* > 0.05). Additionally, compared with the frailty+MetS group, a statistically significant decline of weight, BMI, BFM, FMI, PBF, VFA, along with SMI, ASMI, and FMR were observed in the frailty group (*p <* 0.05), while SVR had a significant increase (*p <* 0.05). The difference of these body composition indicators was not statistically significant between the MetS group and frailty+MetS group (*p* > 0.05). Detailed characteristics are shown in Table 2.

**TABLE 2:** Comparison of body compositions among four groups.

Variables	Frailty (*n* = 45)	MetS (*n* = 58)	Frailty+MetS (*n* = 12)	Normal (*n* = 171)	*F*(*H*)	*p* value
Height (cm)	165.00 (10.8)	**170.00 (8.5)** ^a^	165.50 (12.3)	165.00 (10.5)	**5.723c**	0.001
Weight (kg)	**57.48 ± 7.90** ^a,b^	**74.18 ± 9.69** ^a^	**69.63 ± 10.58** ^a^	62.73 ± 9.28	33.353	<0.001
BMI (kg/m^2^)	**21.30 ± 2.77** ^a,b^	**26.00 ± 2.84** ^a^	**25.88 ± 3.20** ^a^	23.09 ± 2.65	16.292	<0.001
BFM (kg)	**12.28 ± 5.01** ^a,b^	**21.70 ± 5.86** ^a^	**20.12 ± 6.61** ^a^	15.04 ± 5.73	32.838	<0.001
FMI (kg/m^2^)	**4.61 ± 2.04** ^a,b^	**7.67 ± 2.18** ^a^	**7.59 ± 2.70** ^a^	5.58 ± 2.19	23.316	<0.001
PBF	**0.211 ± 0.075** ^a,b^	**0.290 ± 0.060** ^a^	**0.288 ± 0.078** ^a^	0.236 ± 0.075	15.865	<0.001
VFA (cm^2^)	**62.40 (40.60)** ^a,b^	**98.15 (52.95)** ^a^	**94.40 (60.45)** ^a^	67.80 (38.10)	**30.560c**	<0.001
SMM (kg)	**24.52 ± 3.85** ^a^	**28.90 ± 3.90** ^a^	26.93 ± 4.99	26.10 ± 4.23	9.449	<0.001
SMI (kg/m^2^)	**9.04 ± 1.04** ^a,b^	**10.09 ± 0.86** ^a^	9.93 ± 1.16	9.57 ± 0.99	10.339	<0.001
ASMI (kg/m^2^)	**6.75 ± 0.87** ^a,b^	**7.60 ± 0.77** ^a^	7.33 ± 1.01	7.12 ± 0.84	8.180	<0.001
FMR	**0.487 (0.31)** ^b^	**0.708 (0.31)** ^a^	**0.735 (0.52)** ^a^	0.556 (0.35)	**11.764c**	<0.001
SVR	**0.12 (0.07)** ^b^	**0.08 (0.03)** ^a^	**0.08 (0.05)** ^a^	0.10 (0.06)	**7.932c**	<0.001

*Note*: c represents *H* value.

Abbreviations: ASMI, appendicular skeletal muscle mass index; BFM, body fat mass; BMI, body mass index; FMI, fat mass index; FMR, fat‐to‐muscle mass ratio; PBF, percent body fat; SMI, skeletal muscle mass index; SMM, skeletal muscle mass; SVR, skeletal muscle mass to visceral fat area ratio; VFA, visceral fat area. [Correction added on Oct 7, 2024 after first online publication. The superscript in the column ‘Fraility’ has been changed from ‘a’ to ‘ab’ in this version.]

^a^ *p <* 0.05, compared with the N group.

^b^ *p <* 0.05, compared with the FM group.

### 3.3 Detailed occurrence of postoperative complications

As shown in Table 1, there were statistically significant difference in terms of incidence of postoperative total and severe complications among four groups. The incidence of postoperative total complications and severe complications was 19.93% and 7.34%. Specifically, postoperative infection was the most common complication, which accounted for 6.43%, 15.56%, 8.62%, and 33.33% in the normal group, frailty group, MetS group, and frailty+MetS group, respectively. In addition, the incidence of hematological‐, pulmonary‐, and wound‐related complications in the frailty group was 6.67%, 4.44%, and 2.22%, respectively, and 8.89% participants had two or more complications in this group. The incidence of wound and gastrointestinal complications in the frailty+MetS group was both 8.33%. Detailed information about postoperative complications is summarized in Table 3.

**TABLE 3:** Specific occurrence of postoperative complications (*n* %).

Total (*n* = 286)	Frailty (*n* = 45)	MetS (*n* = 58)	Frailty+MetS (*n* = 12)	Normal (*n* = 171)
Total complication (*n* = 57 (19.93%))	17 (37.78%)	9 (15.52%)	6 (50%)	25 (14.62%)
Infectious (*n* = 27 (9.44%))	7 (15.56%)	5 (8.62%)	4 (33.33%)	11 (6.43%)
Wound (*n* = 4 (1.40%))	1 (2.22%)	1 (1.72%)	1 (8.33%)	1 (0.58%)
Gastrointestinal (*n* = 3 (1.05%))	0 (0%)	0 (0%)	1 (8.33%)	2 (1.17%)
Pulmonary (*n* = 3 (1.05%))	2 (4.44%)	0 (0%)	0 (0%)	1 (0.58%)
Hematological (*n* = 11 (3.85%))	3 (6.67%)	1 (1.72%)	0 (0%)	7 (4.09%)
Combine 2 or more (*n* = 9 (3.15%))	4 (8.89%)	2 (3.45%)	0 (0%)	3 (1.75%)
Severe complication (*n* = 21 (7.34%))	9 (20%)	4 (6.90%)	3 (25%)	5 (2.92%)

### 3.4 Regression analysis of postoperative total complications

The result of univariate analysis showed that age (OR = 1.055, 95% CI: 0.996–1.117), comorbidities (OR = 2.427, 95% CI: 1.311–4.494), polypharmacy (OR = 4.440, 95% CI: 1.490–13.228), anemia (OR = 3.083, 95% CI: 1.619–5.870), pTNM III (OR = 1.867, 95% CI: 0.929–3.751), laparoscopic surgery (OR = 0.376, 95% CI: 0.181–0.783), frailty+MetS group (OR = 5.840, 95% CI: 1.744–19.555), and frailty group (OR = 3.546, 95% CI: 1.697–7.409) could be the potential factors of postoperative total complications (*P <* 0.10). Further including these indictors into multivariate analysis, the results showed that comorbidities (OR = 2.322, 95% CI: 1.131–4.767), polypharmacy (OR = 4.527, 95% CI: 1.316–15.574), anemia (OR = 2.765, 95% CI: 1.390–5.503), frailty group (OR = 3.068, 95% CI: 1.402–6.713), and frailty+MetS group (OR = 3.852, 95% CI: 1.020–14.539) were independent risk factors of postoperative total complications (*p <* 0.05). The results indicated that there were no multicollinearities between independent variables as all the VIF were below 5. Detailed information is summarized in Table 4.

**TABLE 4:** Univariate and multivariate Logistic regression analysis of postoperative total complications.

Variables	Univariate analysis OR (95% CI)	*p* value	Multivariate analysis OR (95%CI)	*p* value
Gender				
Male	1.00 (reference)	‐		
Female	1.383 (0.707–2.706)	0.344		
Age (year)	1.055 (0.996–1.117)	0.068		
BMI (kg/m^2^)				
Normal 18.5 ~ <24.0	1.00 (reference)	‐		
Low <18.5	0.591 (0.164–2.139)	0.423		
Overweight 24 ~ <28	0.914 (0.470–1.778)	0.791		
Obese ≥28	0.608 (0.234–1.578)	0.307		
Comorbidity				
No	1.00 (reference)	‐	1.00 (reference)	‐
Yes	2.427 (1.311–4.494)	0.005	2.322 (1.131–4.767)	0.022
Polypharmacy				
No	1.00 (reference)	‐	1.00 (reference)	‐
Yes	4.440 (1.490–13.228)	0.007	4.527 (1.316–15.574)	0.017
History of smoking				
No	1.00 (reference)	‐		
Yes	0.726 (0.399–1.321)	0.295		
History of drinking				
No	1.00 (reference)	‐		
Yes	0.673 (0.368–1.230)	0.198		
History of abdominal surgeries				
No	1.00 (reference)	‐		
Yes	0.749 (0.330–1.700)	0.489		
NRS2002 score ≥3				
No	1.00 (reference)	‐		
Yes	1.034 (0.495–2.161)	0.928		
Hemoglobin, g/L				
Normal	1.00 (reference)	‐	1.00 (reference)	‐
Low	3.083 (1.619–5.870)	0.001	2.765 (1.390–5.503)	0.004
Serum albumin, g/L				
Normal	1.00 (reference)	‐		
Low	1.620 (0.863–3.041)	0.133		
pTNM				
I	1.00 (reference)	‐		
II	1.177 (0.530–2.615)	0.668		
III	1.867 (0.929–3.751)	0.080		
Operation method				
Open surgery	1.00 (reference)	‐		
Laparoscopic surgery	0.376 (0.181–0.783)	0.009		
Tumor size				
<5 cm	1.00 (reference)	‐		
≥5 cm	1.272 (0.694–2.332)	0.437		
Tumor differentiation				
Low	1.00 (reference)	‐		
Moderate	1.214 (0.648–2.276)	0.544		
High	1.132 (0.377–3.395)	0.825		
Operation type				
Total	1.00 (reference)	‐		
Distal	0.625 (0.328–1.190)	0.153		
Proximal	0.386 (0.085–1.745)	0.216		
MetS and frailty				
Normal	1.00 (reference)	‐	1.00 (reference)	‐
Frailty+MetS	5.840 (1.744–19.555)	0.004	3.852 (1.020–14.539)	0.047
MetS	1.073 (0.469–2.454)	0.868	0.670 (0.266–1.684)	0.394
Frailty	3.546 (1.697–7.409)	0.001	3.068 (1.402–6.713)	0.005

Abbreviations: BMI, body mass index; NRS2002, nutritional risk screening 2002; pTNM, pathologic tumor‐node‐metastasis; SD, standard deviation.

### 3.5 Regression analysis of postoperative severe complications

The result of univariate analysis showed that gender (OR = 2.405, 95% CI: 0.949–6.095), age (OR = 1.137, 95% CI: 1.038–1.244), polypharmacy (OR = 3.848, 95% CI: 0.985–15.042), anemia (OR = 2.371, 95% CI: 0.893–6.299), laparoscopic surgery (OR = 0.135, 95% CI: 0.053–0.346), frailty+MetS group (OR = 11.067, 95% CI: 2.278–53.768), and frailty group (OR = 8.300, 95% CI: 2.625–26.241) could be the potential factors of postoperative severe complications (*p <* 0.10). Further including these indictors into multivariate analysis, the results showed that polypharmacy (OR = 4.759, 95% CI: 1.032–21.950), laparoscopic surgery (OR = 0.128, 95% CI: 0.046–0.358), frailty group (OR = OR = 9.423, 95% CI: 2.725–32.589), and frailty+MetS group (OR = 12.096, 95% CI: 2.183–67.024) were independent risk factors of postoperative severe complications (*p <* 0.05). The results indicated that there were no multicollinearities between independent variables as all the VIF were below 5. Detailed information is summarized in Table 5.

**TABLE 5:** Univariate logistic regression analysis of postoperative severe complications.

Variables	Univariate analysis OR (95% CI)	*p* value	Multivariate analysis OR (95% CI)	*p* value
Gender				
Male	1.00 (reference)	‐		
Female	2.405 (0.949–6.095)	0.064		
Age (year)		0.005		
BMI (kg/m^2^)				
Normal 18.5 ~ <24.0	1.00 (reference)	‐		
Low <18.5	‐	0.998		
Overweight 24 ~ <28	0.631 (0.217–1.838)	0.399		
Obese ≥28	0.777 (0.210–2.871)	0.706		
Comorbidity				
No	1.00 (reference)	‐		
Yes	1.985 (0.776–5.075)	0.152		
Polypharmacy				
No	1.00 (reference)	‐	1.00 (reference)	‐
Yes	3.848 (0.985–15.042)	0.053	4.759 (1.032–21.950)	0.046
History of smoking				
No	1.00 (reference)	‐		
Yes	0.803 (0.322–2.001)	0.637		
History of drinking				
No	1.00 (reference)	‐		
Yes	0.652 (0.255–1.668)	0.372		
History of abdominal surgeries				
No	1.00 (reference)	‐		
Yes	0.793 (0.224–2.806)	0.720		
NRS2002 score ≥3				
No	1.00 (reference)	‐		
Yes	1.808 (0.667–4.901)	0.244		
Hemoglobin, g/L				
Normal	1.00 (reference)	‐		
Low	2.371 (0.893–6.299)	0.083		
Serum albumin, g/L				
Normal	1.00 (reference)	‐		
Low	1.893 (0.751–4.771)	0.176		
pTNM				
I	1.00 (reference)	‐		
II	0.869 (0.265–2.850)	0.816		
III	1.146 (0.410–3.202)	0.795		
Operation method				
Open surgery	1.00 (reference)	‐	1.00 (reference)	‐
Laparoscopic surgery	0.135 (0.053–0.346)	<0.001	0.128 (0.046–0.358)	<0.001
Tumor size				
<5 cm	1.00 (reference)	‐		
≥5 cm	1.041 (0.405–2.672)	0.934		
Tumor differentiation				
Low	1.00 (reference)	‐		
Moderate	0.653 (0.250–1.704)	0.384		
High	1.455 (0.364–5.818)	0.596		
Operation type				
Total	1.00 (reference)	‐		
Distal	0.393 (0.128–1.211)	0.104		
Proximal	0.524 (0.066–4.191)	0.543		
MetS and frailty				
Normal	1.00 (reference)	‐	1.00 (reference)	‐
Frailty+MetS	11.067 (2.278–53.768)	0.003	12.096 (2.183–67.024)	0.004
MetS	2.459 (0.637–9.488)	0.191	2.049 (0.492–8.525)	0.324
Frailty	8.300 (2.625–26.241)	<0.001	9.423 (2.725–32.589)	<0.001

Abbreviations: BMI, body mass index; NRS2002, nutritional risk screening 2002; pTNM, pathologic tumor‐node‐metastasis; SD, standard deviation.

## 4 DISCUSSION

In this study, the prevalence of preoperative frailty, MetS, and frailty+MetS was 15.7%, 20.3%, and 4.2% respectively. Compared with the normal group, the frailty group showed an overall decrease in fat‐ and muscle‐related compositions, while the MetS group was characterized by an overall increase in fat‐ and muscle‐related compositions, as well as a significant increased ratio of fat to muscle indicators. In the frailty+MetS group, there was a significant increase in fat‐related compositions and the ratio of fat to muscle indicators, without a significant change in muscle‐related compositions. Additionally, although both fat‐ and muscle‐related compositions in the frailty group decreased significantly compared with the frailty+MetS group, the ratio of muscle to fat indicators increased significantly. Moreover, the findings of this study also suggested that MetS alone had no significant effect on postoperative complications, while frailty alone and frailty combined with MetS were independent risk factors for postoperative complications, with the risk being further increased in the frailty+MetS group compared with the frailty group.

In this study, an overall increase in fat and muscle compositions was observed in the MetS group. A recent survey showed that MetS was greatly related to socioeconomic level and lifestyle behavior, and a higher prevalence of MetS was observed among people from urban, eastern areas, and high‐income families in China.^35^ These indicated a better nutritional intake and may help explain the body compositions characteristics of the MetS group. In this study, MetS alone did not demonstrated a significant association with postoperative complications in this study. This result was similar with Xu et al,^10^ who found that gastric cancer patients combined with MetS alone had a better prognosis than other patients. First, malnutrition can greatly affect postoperative outcome among gastric cancer patients,^36^ while our patients with MetS alone experienced a better nutritional status, manifested as better albumin, hemoglobin, NRS2002 score, and body compositions. Considering the high prevalence of malnutrition in patients undergoing surgery for gastrointestinal cancers, the adequate nutrition reserves could help protect against nutrient loss from tumor‐related treatments and reduce postoperative complications.^37^ Moreover, age was one of the factors influencing the association between MetS and postoperative outcomes in patients with gastric cancer. Chen et al.^38^ found that MetS was an independent risk factor for postoperative complications in young people under 65 years, while no correlation was found in older people over 65 years. This study included older people from 60 to 80 years, and therefore the result should be verified in different age groups in future studies.

By contrast, both fat and muscle compositions of the frailty group were significantly lower in this study. Although the decline in muscle mass and function was generally considered a core feature of frailty,^39^ recently researchers have found that adipose tissue could act as an energy reserve to prevent malnutrition and may have a protective effect on survival among frail people.^17^ Jyväkorpi et al.^40^ demonstrated that frailty was related to lower BMI, lower muscle mass, and poorer nutritional status, and they also found a significantly negative relationship between frailty and fat intake of daily dietary.^41^ However, a significant correlation was merely found between muscle quantity and quality and frailty among patients receiving abdominal surgery in the study of Fumagalli et al., instead of body size and adipose tissue distribution.^42^ In addition to cross‐sectional studies, a longitudinal birth cohort study demonstrated that frail people with a faster decline of lean indices and a faster increase of fat indices and fat‐to‐lean ratios from midlife into old age may experience a worse health outcome.^43^ Currently most research has targeted at the role of protein in the nutrition management of frailty, while few studies focused on fat supplement. Future study could further explore fat metabolism in the pathogenesis of frailty and the synergistic effect of protein and fat, aiming to design a comprehensive, and scientific dietary supplement model to improve the nutritional intake and absorption of frail people.

The result of a meta‐analysis from our research team indicated that preoperative frailty was an important risk factor for postoperative complications among patients with digestive system tumors,^8^ which was consistent with findings of this study. However, although both the frailty group and frailty+ MetS group were assessed by frailty phenotypes, their corresponding pathogenesis may be different, indicating a necessity for targeted strategies of frailty management based on distinct underlying biological cascades.^44^ Wang et al summarized six potential biological processed linked to frailty, including brain changes, endocrine bysregulation, enhanced inflammation, immune dysfunction, metabolic imbalance, and oxidative stress.^45^ Additionally, researchers have found that specific subtypes of physical frailty were associated with functional declines, progression of multimorbidity, and slower recovery among older people.^46^, ^47^ In our study, body composition of the frailty+MetS group was characterized by an increase in fat‐related indicators, which was different from the other groups. Considering the worse tumor staging and differentiation, we suspect the body composition characteristics of this groups may be induced by severer tumor associated metabolic change, and the regulation of tumor metabolism maybe the key intervention for this group.

Except muscle‐ or fat‐related compositions alone, the ratio of the two has been increasingly focused on by researchers, of which FMR and SVR are widely used indicators. A Korean study showed that SVR could be used to assess sarcopenic obesity and was closely related to the occurrence and deterioration of MetS.^32^ A Chinese study also suggested that SVR could be a predictor of shift from metabolically healthy obese individuals to unhealthy phenotypes.^48^ In addition, researchers found that FMR was significantly associated with MetS and insulin resistance,^49^ and it could be non‐linear linked with all‐cause mortality among people with different age and sex.^22^ It is worth noting that the characteristics of FMR and SVR were completely opposite between the frailty and frailty+MetS groups in this study, with the ratio of fat to muscle being increasing greatly in the latter. This may indicate severer metabolic dysfunction in the frailty+MetS group and reflect the vicious cycle of MetS and frailty and helps to understand the worse prognosis of this group. Moreover, considering the MetS alone also presented similar features, it is essential to focus on the causal relationship and development trend between MetS and frailty to prevent MetS from shifting into a frailty state.^50^ Although an important difference of fat to muscle ratio among four groups were observed, the linear relationship and the specific cut‐off value for prognosis are still lacking. Future studies could focus on the predictive value of fat to muscle ratios among different populations and determine the optimal cut‐off value.

Several limitations of this study should be considered. First, this is a single‐center study with limited sample size, only focusing one cancer type and including one postoperative outcome indicator. Especially, the sample sizes in sub‐group analyses were small, which limited the generalization of conclusions. Future studies are needed to validate the results in larger, more diverse populations as well as lengthen the observation time. Furthermore, this study only analyzed the characteristics of body compositions based on cross‐sectional data, which could not reflect their dynamic change. Although MetS alone did not have a significant effect on postoperative complications, its causal relationship with frailty and its susceptibility to metabolic disorders remains to be determined. A prospective longitudinal cohort study is needed to clarify the unknowns.

## 5 CONCLUSION AND IMPLICATIONS

In conclusion, both frailty and MetS coexisting with frailty had adverse effects on postoperative complications, with the latter having the highest risk. Our findings reinforce the importance of providing targeted nutritional or metabolic intervention for frailty with distinct pathogenesis, as the two groups appeared greatly different characteristics in body compositions. Moreover, although MetS alone were not significantly associated with postoperative complications, it is essential to focus on the causal relationship and development trend between MetS and frailty to prevent MetS from shifting into frailty, considering the highest risk in their coexistence state.

## AUTHOR CONTRIBUTIONS

**Xiaoman Jiang:** Conceptualization (lead); data curation (lead); methodology (lead); software (equal); writing – original draft (lead). **Lingyu Ding:** Conceptualization (supporting); data curation (equal); formal analysis (equal). **Yinning Guo:** Data curation (equal); investigation (equal); methodology (equal). **Xueyi Miao:** Data curation (equal); methodology (equal); software (equal). **Kang Zhao:** Formal analysis (equal); methodology (equal); software (equal). **Li Chen:** Data curation (equal); resources (lead). **Shuqin Zhu:** Funding acquisition (supporting); supervision (supporting); validation (equal). **Xinyi Xu:** Methodology (equal); supervision (equal); writing – review and editing (equal). **Qin Xu:** Funding acquisition (lead); project administration (lead); supervision (lead); writing – review and editing (equal).

## FUNDING INFORMATION

This study was supported by three projects: Project “The exploration of trajectories and intervention program of frailty for gastric cancer survivors based on the health ecology theory” supported by National Natural Science Foundation of China (NSFC) (no. 82073407); Project “Studies on Construction of Core Competency Model and Development of Assessment Tool for Nurses of Hospice Care” supported by NSFC (no. 72004099); Project of “Nursing Science” Funded by the Priority Discipline Development Program of Jiangsu Higher Education Institutions (2018, no. 87).

## CONFLICT OF INTEREST STATEMENT

The authors declare that they have no conflict of interest.

## Data Availability

Data will be available upon request for the purposes of data transparency.

## References

[1] Sung H , Ferlay J , Siegel RL , et al. Global cancer statistics 2020: GLOBOCAN estimates of incidence and mortality worldwide for 36 cancers in 185 countries. CA Cancer J Clin. 2021;71(3):209‐249. doi:10.3322/caac.21660 33538338

[2] Maomao C , He L , Dianqin S , et al. Epidemiological trend analysis of gastric cancer in China from 2000 to 2019. Chin J Digest Surg. 2021;20(1):102‐109. doi:10.3760/cma.j.cn115610-20201130-00746

[3] Misawa N , Higurashi T , Tachikawa J , et al. Clinical impact of evaluation of frailty in endoscopic submucosal dissection for early gastric cancer in elderly patients. Geriatr Gerontol Int. 2020;20(5):461‐466. doi:10.1111/ggi.13905 32175690

[4] Caihua Z , Pengpeng L , Longbo G , Hao L . Value of a nomogram based on nutritional risk and sarcopenia on predicting postoperative complications in elderly patients with gastric cancer. Chin J Bases Clin Gen Surg. 2023;30(4):431–436. https://link.cnki.net/urlid/51.1505.R.20230315.0903.002

[5] Fried LP , Tangen CM , Walston J , et al. Frailty in older adults: evidence for a phenotype. J Gerontol A Biol Sci Med Sci. 2001;56(3):M146‐M156. doi:10.1093/gerona/56.3.m146 11253156

[6] Boccardi V , Mecocci P . The importance of cellular senescence in frailty and cardiovascular diseases. Adv Exp Med Biol. 2020;1216:79‐86. doi:10.1007/978-3-030-33330-0_9 31894549

[7] Martemucci G , Fracchiolla G , Muraglia M , Tardugno R , Dibenedetto RS , D'Alessandro AG . Metabolic syndrome: a narrative review from the oxidative stress to the management of related diseases. Antioxidants (Basel). 2023;12(12):2091. doi:10.3390/antiox12122091 38136211 PMC10740837

[8] Ding L , Lu J , Zhu H , et al. Effects of preoperative frailty on outcomes following surgery among patients with digestive system tumors: a systematic review and meta‐analysis. Eur J Surg Oncol. 2021;47:3040‐3048. doi:10.1016/j.ejso.2021.07.019 34325940

[9] Jiang X , Xu X , Ding L , et al. The association between metabolic syndrome and presence of frailty: a systematic review and meta‐analysis. Eur Geriatr Med. 2022;13(5):1047‐1056. doi:10.1007/s41999-022-00688-4 36036343

[10] Xu LB , Zhang HH , Shi MM , et al. Metabolic syndrome‐related sarcopenia is associated with worse prognosis in patients with gastric cancer: a prospective study. Eur J Surg Oncol. 2020;46(12):2262‐2269. doi:10.1016/j.ejso.2020.07.032 32800596

[11] Wei XL , Qiu MZ , Lin HX , et al. Patients with old age or proximal tumors benefit from metabolic syndrome in early stage gastric cancer. PLoS One. 2014;9(3):e89965. doi:10.1371/journal.pone.0089965 24599168 PMC3943843

[12] Kane AE , Gregson E , Theou O , Rockwood K , Howlett SE . The association between frailty, the metabolic syndrome, and mortality over the lifespan. Geroscience. 2017;39(2):221‐229. doi:10.1007/s11357-017-9967-9 28281219 PMC5411368

[13] Hao Q , Song X , Yang M , Dong B , Rockwood K . Understanding risk in the oldest old: frailty and the metabolic syndrome in a Chinese community sample aged 90+ years. J Nutr Health Aging. 2016;20(1):82‐88. doi:10.1007/s12603-016-0680-7 26728938

[14] Qichen Z , Jie C . Research progress of frailty, metabolic syndrome and intestinal flora. Chin J Gerontol. 2018;38(19):4837‐4841. https://kns.cnki.net/kcms2/article/abstract?v=mRajgomPiini6BblbNrN83MMBiTcSJP9hn0PjgPkkWt667NZDiGC2VE0l1P7FMu_glAa334a1BIBkb90lqnJu0VOia7PdVHorPAYEbkM7kNj_JGMT5twNKoXPZjW3tknUCJUkrhTtCA2XjKwkvrRdA==&uniplatform=NZKPT&language=CHS

[15] Calvani R , Marini F , Cesari M , et al. Biomarkers for physical frailty and sarcopenia: state of the science and future developments. J Cachexia Sarcopenia Muscle. 2015;6(4):278‐286. doi:10.1002/jcsm.12051 26675566 PMC4670735

[16] Misra A , Soares MJ , Mohan V , et al. Body fat, metabolic syndrome and hyperglycemia in South Asians. J Diabetes Complicat. 2018;32(11):1068‐1075. doi:10.1016/j.jdiacomp.2018.08.001 30115487

[17] Yuan L , Chang M , Wang J . Abdominal obesity, body mass index and the risk of frailty in community‐dwelling older adults: a systematic review and meta‐analysis. Age Ageing. 2021;50(4):1118‐1128. doi:10.1093/ageing/afab039 33693472

[18] Watanabe D , Yoshida T , Watanabe Y , Yamada Y , Miyachi M , Kimura M . Frailty modifies the association of body mass index with mortality among older adults: Kyoto‐Kameoka Study. Clin Nutr. 2024;43(2):494‐502. doi:10.1016/j.clnu.2024.01.002 38184941

[19] Song P , Han P , Zhao Y , et al. Muscle mass rather than muscle strength or physical performance is associated with metabolic syndrome in community‐dwelling older Chinese adults. BMC Geriatr. 2021;21(1):191. doi:10.1186/s12877-021-02143-8 33740914 PMC7980667

[20] Mengzhen W . Relationship between sarcopenic obesity and metabolic syndrome in elderly hospitalized patients. Mater Dissertation Research Paper. 2019;2:49. doi:10.27262/d.cnki.gqdau.2019.001684

[21] Sandini M , Paiella S , Cereda M , et al. Independent effect of fat‐to‐muscle mass ratio at bioimpedance analysis on long‐term survival in patients receiving surgery for pancreatic cancer. Front Nutr. 2023;10:1118616. doi:10.3389/fnut.2023.1118616 37384108 PMC10298166

[22] Yu B , Sun Y , Du X , et al. Age‐specific and sex‐specific associations of visceral adipose tissue mass and fat‐to‐muscle mass ratio with risk of mortality. J Cachexia Sarcopenia Muscle. 2023;14(1):406‐417. doi:10.1002/jcsm.13142 36447372 PMC9891960

[23] Kamarajah SK , Bundred J , Tan BHL . Correction to: body composition assessment and sarcopenia in patients with gastric cancer: a systematic review and meta‐analysis. Gastric Cancer. 2019;22(3):645‐650. doi:10.1007/s10120-019-00939-7 30825006

[24] Xu XY , Jiang XM , Xu Q , et al. Skeletal muscle change during neoadjuvant therapy and its impact on prognosis in patients with gastrointestinal cancers: a systematic review and meta‐analysis. Front Oncol. 2022;12:892935. doi:10.3389/fonc.2022.892935 35692760 PMC9186070

[25] Jiang X , Xu X , Ding L , et al. Preoperative low absolute and relative handgrip strength as predictors of postoperative short‐term outcomes: a prospective study based on patients aged 60 years and older with gastric cancer. Eur Geriatr Med. 2023;14(2):251‐262. doi:10.1007/s41999-023-00768-z 36949226

[26] Chinese Diabetes Society . Guideline for the prevention and treatment of type 2 diabetes mellitus in China (2020 edition). Chin J Diabetes. 2021;13(4):315‐409. doi:10.3760/cma.j.cn115791-20210221-00095

[27] Jie Z , Zhenyun W , Ge F , et al. Development of the Chinese age norms of the CES‐D in urban area. Chin Ment Health J. 2010;24(2):139‐143. doi:10.3969/j.issn.1000-6729.2010.02.015

[28] Lanfang L . Reliability and validity of retrospective physical activity questionnaire in Taizhou population. Mater Dissertation Research Paper; 2011 https://kns.cnki.net/kcms2/article/abstract?v=mRajgomPiinj8168W2wRW22c9m54CxagK3bYOMTTtmrotkTWtzqaawKVwsTfQL65ZqwooLaklmrvc1BjCqr25NCXK7iY8IgvioknN_eXB5oYJo8sgbZoCzXStYwtJkNDM0UBxM8q1f1LsEGoJc8mxQ==&uniplatform=NZKPT&language=CHS

[29] Geriatric Medicine Branch of Chinese Medical Association . Chinese experts consensus on assessment and intervention for elderly patients with frailty. Chinese J Geriat. 2017;36(3):251‐256. doi:10.3760/cma.j.issn.0254-9026.2017.03.007

[30] Kurinami N , Sugiyama S , Nishimura H , et al. Clinical factors associated with initial decrease in body‐fat percentage induced by add‐on sodium‐glucose co‐transporter 2 inhibitors in patient with type 2 diabetes mellitus. Clin Drug Investig. 2018;38(1):19‐27. doi:10.1007/s40261-017-0580-6 PMC5762773 29098566

[31] He H , Pan L , Wang D , et al. Fat‐to‐muscle ratio is independently associated with hyperuricemia and a reduced estimated glomerular filtration rate in Chinese adults: the China National Health Survey. Nutrients. 2022;14(19):4193. doi:10.3390/nu14194193 36235845 PMC9573307

[32] Kim TN , Park MS , Lim KI , et al. Skeletal muscle mass to visceral fat area ratio is associated with metabolic syndrome and arterial stiffness: the Korean Sarcopenic Obesity Study (KSOS). Diabetes Res Clin Pract. 2011;93(2):285‐291. doi:10.1016/j.diabres.2011.06.013 21752483

[33] Clavien PA , Sanabria JR , Strasberg SM . Proposed classification of complications of surgery with examples of utility in cholecystectomy. Surgery. 1992;111(5):518‐526. http://pubmed‐ncbi‐nlm‐nih‐gov‐s.webvpn.njmu.edu.cn:8118/1598671/ 1598671

[34] Grocott MPW , Browne JP , Van der Meulen J , et al. The postoperative morbidity survey was validated and used to describe morbidity after major surgery. J Clin Epidemiol. 2007;60(9):919‐928. doi:10.1016/j.jclinepi.2006.12.003 17689808

[35] Huimin Y , Mei Z , Xiao Z , et al. Study of epidemiological characteristics of metabolic syndrome and influencing factors in elderly people in China. Chinese J Geriat. 2019;40(3):284‐289. doi:10.3760/cma.j.issn.0254-6450.2019.03.006 30884605

[36] Hua H , Xu X , Tang Y , Ren Z , Xu Q , Chen L . Effect of sarcopenia on clinical outcomes following digestive carcinoma surgery: a meta‐analysis. Support Care Cancer. 2019;27(7):2385‐2394. doi:10.1007/s00520-019-04767-4 30955115

[37] Impact of malnutrition on early outcomes after cancer surgery: an international, multicentre, prospective cohort study. Lancet Glob Health. 2023;11(3):e341‐e349. doi:10.1016/S2214-109X(22)00550-2 36796981

[38] Chen X , Zhang W , Sun X , et al. Metabolic syndrome predicts postoperative complications after gastrectomy in gastric cancer patients: development of an individualized usable nomogram and rating model. Cancer Med. 2020;9(19):7116‐7124. doi:10.1002/cam4.3352 33470549 PMC7541147

[39] Nascimento CM , Ingles M , Salvador‐Pascual A , Cominetti MR , Gomez‐Cabrera MC , Viña J . Sarcopenia, frailty and their prevention by exercise. Free Radic Biol Med. 2019;132:42‐49. doi:10.1016/j.freeradbiomed.2018.08.035 30176345

[40] Jyväkorpi SK , Lindström M , Suominen MH , et al. Relationship between frailty, nutrition, body composition, quality of life, and gender in institutionalized older people. Aging Clin Exp Res. 2022;34(6):1357‐1363. doi:10.1007/s40520-022-02077-0 35146701 PMC9151503

[41] Jyväkorpi SK , Urtamo A , Strandberg TE . Dietary fat composition and frailty in oldest‐old men. J Am Geriatr Soc. 2020;68(6):1346‐1348. doi:10.1111/jgs.16402 32142157

[42] Fumagalli IA , Le ST , Peng PD , et al. Automated CT analysis of body composition as a frailty biomarker in abdominal surgery. JAMA Surg. 2024;159(7):766‐774. doi:10.1001/jamasurg.2024.0628 38598191 PMC11007659

[43] Haapanen MJ , Kananen L , Mikkola TM , et al. Frailty in midlife as a predictor of changes in body composition from midlife into old age: a longitudinal birth cohort study. Gerontology. 2024;70(8):831‐841. doi:10.1159/000539204 38718772 PMC11309068

[44] Angioni D , Macaron T , Takeda C , et al. Can we distinguish age‐related frailty from frailty related to diseases ? Data from the MAPT study. J Nutr Health Aging. 2020;24(10):1144‐1151. doi:10.1007/s12603-020-1518-x 33244575

[45] Wang J , Maxwell CA , Yu F . Biological processes and biomarkers related to frailty in older adults: a state‐of‐the‐science literature review. Biol Res Nurs. 2019;21(1):80‐106. doi:10.1177/1099800418798047 30198309

[46] Baldwin MR , Pollack LR , Friedman RA , et al. Frailty subtypes and recovery in older survivors of acute respiratory failure: a pilot study. Thorax. 2021;76(4):350‐359. doi:10.1136/thoraxjnl-2020-214998 33298583 PMC8187474

[47] Huang ST , Tange C , Otsuka R , et al. Subtypes of physical frailty and their long‐term outcomes: a longitudinal cohort study. J Cachexia Sarcopenia Muscle. 2020;11(5):1223‐1231. doi:10.1002/jcsm.12577 32558267 PMC7567152

[48] Guilan C . Association between skeletal muscle mass to visceral fat area ratio and metabolic phenotype of obesity. Mater Dissertation Research Paper. 2020;1:73. doi:10.27003/d.cnki.gojyu.2020.000326

[49] Seo YG , Song HJ , Song YR . Fat‐to‐muscle ratio as a predictor of insulin resistance and metabolic syndrome in Korean adults. J Cachexia Sarcopenia Muscle. 2020;11(3):710‐725. doi:10.1002/jcsm.12548 32030917 PMC7296262

[50] Tamura Y , Omura T , Toyoshima K , Araki A . Nutrition Management in Older Adults with diabetes: a review on the importance of shifting prevention strategies from metabolic syndrome to frailty. Nutrients. 2020;12(11):3367. doi:10.3390/nu12113367 33139628 PMC7693664

