# Correction to “Association of Metabolic Syndrome and Frailty With Postoperative Complications in Older Gastric Cancer Patients: A Body Composition Perspective”

**DOI:** 10.1002/cam4.70380

**Published:** 2024-10-29

Jiang, Xiaoman , Lingyu Ding , Yinning Guo , Xueyi Miao , Kang Zhao , Li Chen , Shuqin Zhu , Xinyi Xu and Qin Xu. 2024. “Association of Metabolic Syndrome and Frailty With Postoperative Complications in Older Gastric Cancer Patients: A Body Composition Perspective.” Cancer Medicine 13(18): e70194. 10.1002/cam4.70194.39315666 PMC11420831

In Table 2 of the Frailty group, the superscripts of weight, BMI, BFM, FMI, PBF, VFA, SMI, and ASMI were incorrect. These superscripts should have been **ab**, which indicated that there existed a significant difference between frailty group and normal group as well as Frailty+MetS group.

We apologize for this error.

